# Constraining the role of early land plants in Palaeozoic weathering and global cooling

**DOI:** 10.1098/rspb.2015.1115

**Published:** 2015-08-22

**Authors:** Joe Quirk, Jonathan R. Leake, David A. Johnson, Lyla L. Taylor, Loredana Saccone, David J. Beerling

**Affiliations:** Department of Animal and Plant Sciences, University of Sheffield, Sheffield S10 2TN, UK

**Keywords:** arbuscular mycorrhiza, atmospheric CO_2_, biological weathering, land plant evolution, Palaeozoic climate

## Abstract

How the colonization of terrestrial environments by early land plants over 400 Ma influenced rock weathering, the biogeochemical cycling of carbon and phosphorus, and climate in the Palaeozoic is uncertain. Here we show experimentally that mineral weathering by liverworts—an extant lineage of early land plants—partnering arbuscular mycorrhizal (AM) fungi, like those in 410 Ma-old early land plant fossils, amplified calcium weathering from basalt grains threefold to sevenfold, relative to plant-free controls. Phosphate weathering by mycorrhizal liverworts was amplified 9–13-fold over plant-free controls, compared with fivefold to sevenfold amplification by liverworts lacking fungal symbionts. Etching and trenching of phyllosilicate minerals increased with AM fungal network size and atmospheric CO_2_ concentration. Integration of grain-scale weathering rates over the depths of liverwort rhizoids and mycelia (0.1 m), or tree roots and mycelia (0.75 m), indicate early land plants with shallow anchorage systems were probably at least 10-fold less effective at enhancing the total weathering flux than later-evolving trees. This work challenges the suggestion that early land plants significantly enhanced total weathering and land-to-ocean fluxes of calcium and phosphorus, which have been proposed as a trigger for transient dramatic atmospheric CO_2_ sequestration and glaciations in the Ordovician.

## 1. Background

Fossil spore assemblages suggest multicellular photosynthetic plants arrived on land sometime in the Ordovician around 470 Ma [1]. However, the effects of these organisms, and the early land floras that subsequently evolved, on the biogeochemical cycling of elements and Ordovician climate are poorly understood and constrained. Nevertheless, plant-driven weathering of silicate minerals is increasingly well understood in terms of the ‘carbon-energy flux’ hypothesis, whereby allocation of photosynthate belowground into roots and associated mycorrhizal fungal networks controls weathering processes [2,3]. These effects are regulated by the productivity and biomass of plants, their evolutionary progression and atmospheric CO_2_ feedbacks over geological timescales [2–4]. In the Ordovician, terrestrial plant productivity and biomass was minimal compared with today, limiting the carbon-energy flux exported belowground and the capacity for plant-related impacts on weathering. Pre-vascular plants may have increased the respiratory generation of CO_2_ and carbonic acid in shallow soils [5,6], but some palaeosol evidence suggests no significant terrestrial biosphere weathering effects prior to the evolution of vascular plants [7].

Numerical modelling of annual photosynthetic CO_2_ fixation by lichens and bryophytes (mosses, hornworts and liverworts) provides insight into the potential rock weathering rates required to mobilize the inorganic phosphate they need to grow. Modelled contemporary global net carbon uptake by lichens and bryophytes ranges from 300 to 3000 Tg yr^−1^ (1 Tg = 1 × 10^12^ g) with an implied phosphate uptake translating into regional rock weathering rates of 1–5 cm^3^ m^−2^ of colonized land yr^−1^ [8,9]. These fluxes agree with calculated field estimates for weathering in an Icelandic basaltic catchment sparsely covered with non-vascular plants and lichens (≈2 cm^3^ m^−2^ yr^−1^). Such fluxes are threefold lower than those under dwarf birch trees (≈6 cm^3^ m^−2^ yr^−1^) in a neighbouring catchment of the same basaltic lithology and climate [10].

Experimental studies investigating the effects of non-vascular plants on weathering are limited. In microcosm experiments comparing a thalloid liverwort (*Conocephalum conicum*), a non-rooted tracheophyte (*Psilotum nudum*) and a rooted tracheophyte (*Equisetum hyemale*) grown in mixed sand and peat substrates under ambient and elevated CO_2_ concentrations, only *Equisetum* enhanced soil solution *p*CO_2_ [6]. In a closed-system laboratory experiment, protonemata of the highly derived moss, *Physcomitrella*, amplified Ca (1.4–3.6 times) and Mg (1.5–5.4 times) dissolution from 15 mm beds of granite or andesite grains and were estimated to increase P release from granite 58-fold, compared with moss-free, sterile conditions [11]. Field evidence from boreal forests in northwestern Ontario suggests lichens and mosses cause intense chemical weathering leading to the production of secondary minerals, clays and thin soils; these effects being absent from adjacent bare areas of the same granitic outcrop [12]. However, the oldest unambiguous fossil mosses date to the Carboniferous (330 Ma) [13] and morphologically, extant mosses possess sophisticated multicellular rhizoids that universally lack fungal symbionts. By contrast, symbioses with mycorrhizal fungi were well established in land plants by 410 Ma [14], apparently having co-evolved with their earliest ancestors over 450 Ma [15,16], and have recently been found to play a direct role in mineral weathering under modern forest trees [4].

Understanding the role of early land plants in Earth's Ordovician environmental history, therefore, requires experiments using extant early land plant lineages with simple rhizoids and fungal symbionts to resolve their effects on biological weathering [17]. Here we report controlled-environment experiments investigating the physical and chemical weathering of silicate rocks and minerals by liverworts, a non-vascular lineage with close affinities to the earliest Ordovician land plant fossils [18]. Liverworts form mutualistic mycorrhiza-like associations that are highly adapted to mobilize mineral P via hyphal networks, which transfer water and nutrients to support plant growth in exchange for photosynthate [19,20]. We conducted experiments with the dominant haploid gametophyte life cycle stage of the simple thalloid liverwort, *Marchantia paleacea* (electronic supplementary material, figure S1) grown in free-draining substrate alongside plant-free controls. To isolate the direct effects of their arbuscular mycorrhiza-like, (AM) glomeromycotean fungal partners, liverworts were grown with (mycorrhizal, M) or without (non-mycorrhizal, NM) fungal symbionts. Uniform grains of basalt were buried beneath both sets of plants in mesh bags accessible to rhizoids and AM fungal hyphae. Basalt is rich in Ca-silicates and plays a central role in the geochemical carbon cycle [21]. We investigated the potential feedbacks of CO_2_ on weathering by conducting our experiments at three atmospheric CO_2_ concentrations representing Palaeozoic (1200 ppm), modern (450 ppm) and Earth's minimum glacial (200 ppm) atmospheric concentrations.

## 2. Material and methods

### (a) Experimental details

We established colonies of *M. paleacea* (Bertol.) from non-mycorrhizal gemmae, half of which were inoculated with their natural mycorrhizal partners by growing them alongside mycorrhizal adult plants. DNA sequencing of the fungal endosymbionts (described previously [20]) confirmed the liverworts were partnered by Glomeromycotean fungi. The experimental plants were grown in free-draining containers on pure quartz substrate mixed with 2.7% dwt *Sphagnum* peat, with plant-free treatments maintained in parallel. All containers were misted daily with deionized water to maintain moist conditions. Well-characterized Tertiary basalt grains (2 g of 0.25–1.00 mm diameter, X-ray fluorescence data given in [4]) were buried 1–2 cm deep within porous mesh bags (two per container; 35 µm pore size) allowing rhizoids and fungal hyphae to interact with the basalt. We maintained experiments for 12 months in controlled-environment growth chambers at 1200, 450 and 200 ppm CO_2_. Overall, *n* = 5 colonies of *M. paleacea* per mycorrhizal type (M, NM and plant-free controls) per CO_2_ treatment.

### (b) Hyphal colonization of basalt

Basalt grains were recovered from mesh bags and 1 g sub-samples were sonicated in 30 ml of deionized water to release hyphae into suspension. Aliquots (4 ml) were filtered onto a membrane, and the retained hyphal fragments stained and hyphal lengths quantified at 200 × magnification using line-intersect counts. Hyphal lengths found in NM and plant-free treatments were ascribed to saprotrophic fungi. AM hyphal lengths were determined by subtracting hyphal lengths found in plant-free control treatments.

### (c) Element uptake into liverworts

Homogenized tissue was digested in 3 : 1 HCl : HNO_3_ at 150°C. Residues were diluted and Ca concentrations determined using inductively coupled plasma mass spectrometry (ICP-MS). Phosphorus content of thallus tissue was determined spectrophotometrically with a standard ammonium molybdate colour-development assay following H_2_SO_4_ digestion at 365°C and clearing with H_2_O_2_.

### (d) Mineral surface alteration

Flakes of biotite and phlogopite embedded in silicone and mounted on glass slides were co-buried in the mesh bags with basalt [22]. Biotite and phlogopite are Fe- and Mg-bearing phyllosilicates, respectively, and ideal for micro-topographic surface characterization with vertical scanning interferometry (VSI) because they have near-atomically smooth cleavage surfaces. We characterized the surface roughness of two specific areas on each mineral flake, in each mesh bag at 500× magnification using VSI before and after incubation in experimental treatments. Mineral surface alteration was quantified by post- to pre-burial ‘roughness ratios’, where values >1 indicate surface roughness increases following physical alteration [22]. Roughness ratios were averaged to give one value per mineral type, per replicate liverwort colony (*n* = 5 colonies). Where etching and trenching of mineral surfaces by fungal hyphae was observed in M liverwort treatments, nano-scale measurements of trench width and depth were made using VSI to calculate trench cross-sectional areas (mm^2^) (*n* = 3–5).

### (e) Mineral dissolution

Calcium dissolution from basalt was determined by subjecting grains from the experimental treatments and freshly prepared, unweathered grains to sequential chemical extractions of exchangeable, trace carbonate and oxide fractions [2,4]. Extraction solutions were analysed for Ca using ICP-MS and phosphate was quantified spectrophotometrically, as above. Calcium and P dissolution was calculated for the carbonate and oxide fractions, relative to unweathered basalt grains (electronic supplementary material, eq. (S1))—or plant-free treatments (electronic supplementary material, eq. (S2)) for comparisons with trees—and summed along with Ca or P taken-up into biomass to give weathering amplification factors over plant-free conditions.

### (f) Comparisons between liverwort, lichen and moss weathering

Calcium dissolution rates (µmol g^−1^ rock yr^−1^) from liverwort treatments were converted to volumetric rock weathering using the concentration of Ca in the basalt (5.4 mmol Ca cm^−3^) assuming a basalt density of 2.9 g cm^−3^ (electronic supplementary material, table S1). We then derived rock volume weathering rates from the literature for comparison with our observations. Weathering potential (WP) was calculated for the liverworts, lichens and mosses, using the P-uptake required by the organisms to grow (µmol m^−2^ land yr^−1^), and rock weathering (cm^3^ m^−2^ land yr^−1^) by the organisms was calculated after accounting for resorption and leaching of P (after [9]). Annual P requirement for the liverworts was calculated from thallus growth and P-content, and for lichens and mosses, using P contents and growth rates reported in the literature (electronic supplementary material, Detailed methods). We then calculated the potential of the organisms to weather basalt using the P concentration of our basalt (0.13 mmol P cm^−3^ assuming a density of 2.8 g cm^−3^) (electronic supplementary material, table S1). Finally, we compiled direct observations of weathered rock volumes on alpine moraines [23] and basalt lava flows [24] of known ages with and without lichen colonization that included observations of olivine and plagioclase weathering and porosity [24]. Annual increases in mineral porosity were converted into mass loss based on the density of olivine (3.8 g cm^−3^) and plagioclase (2.7 g cm^−3^) and the reported depth of the weathering rinds beneath lichens (50 µm) [24]. Subsequently, basalt weathering was calculated based on measured proportions of olivine and plagioclase in basalt (electronic supplementary material, table S1).

### (g) Scaling and comparison with tree weathering

Trees typically have rooting systems around 0.75 m deep [25], whereas the rhizoids and mycorrhizal fungi of liverworts typically penetrate to depths of approximately 0.1 m [26]. These rhizoid/rooting depths are used to calculate the soil volume under the influence of mycorrhizal hyphae (the ‘hyphosphere’) beneath 1 m^2^ of either liverworts or trees. Following published measurements [26], we inferred mycorrhizal hyphae extend to a depth of 12 cm for liverworts and defined a hyphal length density of 5 m cm^−3^ soil, and a hyphosphere radius of five times mean hypha radius (1.4 µm) [27,28]. The hyphosphere is defined as a hollow cylindrical volume: *l* · *π* · (*H*_'sphere_^2^—*H*^2^), where *l* is the length of hyphae, *H*_'sphere_ is hyphosphere radius and *H* is mean hyphal radius. Scaling against observed hyphal length densities colonizing basalt grains in each treatment, we calculated a hyphosphere volume for each individual liverwort colony (mean = 4.4 × 10^−5^ m^3^ m^−2^ land). This will tend to overestimate weathering rates as rhizoid and hyphal lengths are likely to decrease with depth to 12 cm.

Species of saplings used for comparison with liverworts included the AM gymnosperms, *Ginkgo biloba* and *Sequoia sempervirens* and ectomycorrhizal (EM) gymnosperm, *Pinus sylvestris*; and the AM and EM angiosperms, *Magnolia grandiflora* and *Betula pendula*, respectively [2]. Trees (*n* = 4 individuals per species per CO_2_ treatment) were grown in sand and compost alongside plant-free treatments at either 450 or 1500 ppm CO_2_ for approximately six months. The basalt grains and sequential extraction method for determining Ca dissolution were identical to this study [2]. We estimated typical mycorrhizal hyphosphere volumes for each tree species in the field by assuming that the distributions of fine roots and mycorrhizal hyphae typically decline exponentially with depth [25,29]. Fine root distributions of trees are a function of net primary productivity (NPP; g C m^−2^ yr^−1^) and root length density at the top of the soil [25,27,28]. Mycorrhizal fungi follow a similar distribution, which can be integrated over the soil profile to yield the total length of hyphae per m^2^ land [28] (see the electronic supplementary material, Detailed methods). Mean calculated hyphosphere volume for the trees using typical hyphal length densities at the top of the soil (5 m cm^−3^ for AM fungi and approx. 250 m cm^−3^ for EM fungi) was 303 × 10^−5^ m^3^ m^−2^ land. Using hyphal length measurements colonizing basalt grains for both liverworts and trees, we determined Ca-silicate weathering rates per unit hyphosphere volume, which were scaled-up to the soil profile to give Ca-weathering fluxes per m^2^ land, assuming a soil calcium molar fraction of 0.5% [10,30].

### (h) Statistics

Two-way ANOVAs testing for effects of mycorrhiza and CO_2_ were conducted on hyphal length, pH, mineral roughness and calcium and phosphorus weathering rates (plus tissue contents) and amplification factors. Trench dimensions were tested for effects of mineral type and CO_2_. ANCOVAs were run with pH as a covariate to verify that calcium weathering amplification was not driven by bulk pH effects.

## 3. Results and discussion

**Figure 1.**
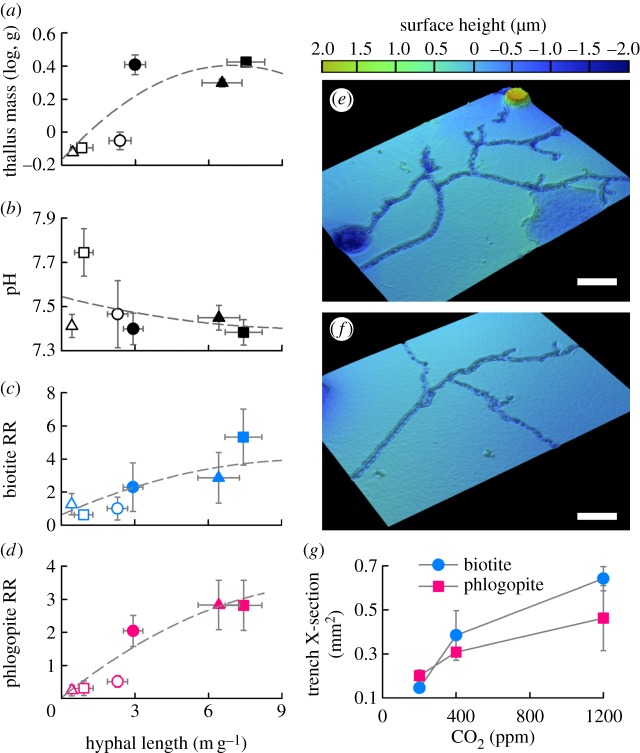
Increasing hyphal network size drives greater mineral surface alteration. Relationship between hyphal length network size colonizing basalt grains and (*a*) liverwort thallus biomass, (*b*) pH of basalt bag solutions, (*c,d*) mineral surface alteration as determined by before-and-after surface roughness ratios (RR) for biotite and phlogopite. Circles are 200 ppm, squares are 450 ppm and triangles are 1200 ppm CO_2_; error bars show s.e.m. Mineral surface micro-topography revealing trenching of (*e*) phlogopite and (*f*) biotite at 1200 ppm CO_2_. Surface height is expressed relative to the mean surface plane, which is equal to zero. Scale bars are approx. 20 µm. (*g*) Analysis of trench morphology revealed that fungal trench size increased with CO_2_. Closed symbols are plants with AM fungal symbionts, open symbols are plants without these fungal partners.

Mycorrhizal hyphal networks extended from the M liverworts to actively colonize basalt grains in the mesh bags immediately beneath them (figure 1). Hyphal densities for the liverworts (3–7 m g^−1^) were similar to those reported beneath AM trees in pot studies [22] (1–3 m g^−1^) and field studies [4] (2–4 m g^−1^). After 12 months, M liverworts grew significantly larger than NM liverworts (*F*_1,24_ = 35.81; *p* < 0.0001; figure 1*a*), demonstrating the physiological benefits of symbiotic fungi that provide weathered nutrients to host plants [20]. Greater hyphal colonization of basalt for M liverworts corresponded with acidification of bulk soil water chemistry compared with NM and plant-free treatments (*F*_2,36_ = 19.36; *p* < 0.0001; figure 1*b*). Hyphal networks were significantly larger at ambient and elevated CO_2_ compared with glacial CO_2_ concentrations (mycorrhiza × CO_2_: *F*_2,24_ = 18.25; *p* < 0.0001), as also reported for trees observed in similar experiments across the same CO_2_ range [22].

Mineral surface VSI scans on flakes of biotite and phlogopite beneath M liverworts revealed distinctive fungal trenching similar to that generated under AM trees in laboratory experiments [22]. Topographic analyses revealed branching linear grooves (2–3 µm wide) etched into biotite and phlogopite that were morphologically consistent with Glomeromycotean fungal colonization of the minerals (figure 1*e,f*). Delamination of phyllosilicate layers (approx. 0.5 µm deep) occurred along the axes of hyphal trenches (lower-right corner of figure 1*e*), with similarities to physical disruption of biotite by EM fungi of pine seedlings [31]. Mycorrhizal liverworts amplified mineral roughness increases over NM liverworts up to eightfold for biotite (*F*_1,24_ = 6.9; *p* = 0.015) and 11-fold for phlogopite (*F*_1,24_ = 29.8; *p* < 0.0001; figure 1*c,d*). This effect was linked to larger mycorrhizal hyphal network sizes compared with the saprotrophic-only fungal networks in NM treatments. Increases in the roughness of mineral flakes beneath M liverworts are comparable with those reported using similar experimental protocols under temperate mycorrhizal trees [22]. At glacial CO_2_ concentrations, hyphal trench cross-sectional areas decreased on both phlogopite (1.6-fold) and biotite (2.5-fold) (*F*_2,10_ = 6.48; *p* = 0.016) relative to 450 ppm and 1200 ppm (figure 1*g*), suggesting direct mineral alteration by liverworts is mediated by enhanced photosynthate supply to mycorrhiza with rising atmospheric CO_2_, as it is for trees [22].

**Figure 2.**
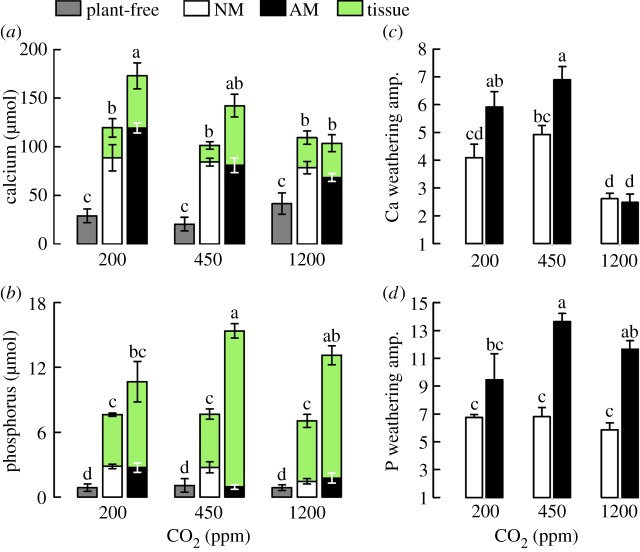
Enhanced grain-scale basalt weathering by liverworts. Weathered amounts of (*a*) calcium and (*b*) phosphorus from basalt grains, including uptake into liverwort tissues (mean ± s.e.m.). Amplification of (*c*) calcium and (*d*) phosphorus weathering (including uptake into liverwort tissues) from basalt grains relative to plant-free treatments at each growth CO_2_ concentration (mean ± s.e.m.). Bars or stacks sharing the same letter are not significantly different at *α* = 0.05 (two-way ANOVA with *post hoc* Tukey tests).

We determined Ca and P weathering from basalt grains by M and NM liverworts, relative to plant-free controls. Calcium is important for multi-million-year atmospheric CO_2_ regulation [11,32], and P for marine productivity and organic carbon burial [11,33]. Dissolution of Ca and P beneath liverworts was significantly higher compared with plant-free controls (Ca: *F*_2,36_ = 83.7; *p* < 0.0001. P: *F*_2,36_ = 148.5; *p* < 0.0001; figure 2*a,b*), but was unrelated to bulk solution pH for both Ca (*F*_1,35_ = 0.02; *p* = 0.9) and P (*F*_1,23_ = 0.03; *p* = 0.9). Elemental uptake into liverwort biomass represented a substantial sink for Ca and P released by weathering. The strongest effect of the symbiosis occurred at 450 ppm CO_2_, under which M liverworts took up over three times more Ca and P compared with their NM counterparts (figure 2*a,b*). Elements were predominantly derived from basalt, the largest source of mineral nutrients in our experiments (approx. 400 µmol total P; approx. 5500 µmol total Ca; electronic supplementary material, table S1); organic matter constituted 2.7% dwt of the substrate (electronic supplementary material, Detailed methods).

Overall, M liverworts amplified grain-scale Ca weathering from basalt over plant-free controls to an extent comparable with NM liverworts—a factor of 2.5–7.0 (figure 2*a,c*). These results compare with 1.4–3.6-fold Ca weathering amplification from a shallow bed of granite and andesite grains reported for the moss, *Physcomitrella* [11]. In contrast to Ca weathering, M liverworts strongly amplified P weathering from basalt grains (13-fold amplification) over NM liverworts (sevenfold) (*F*_1,24_ = 46.13; *p* < 0.0001), reflecting the importance of AM in P uptake (figure 2*b,d*). This benefit of AM fungal weathering was most marked at atmospheric CO_2_ concentrations above 200 ppm (figure 2*d*), in agreement with the carbon-energy flux hypothesis whereby higher atmospheric CO_2_ promotes photosynthesis and growth [20] and intensifies weathering [2]. However, P-weathering amplification of basalt grains by M liverworts was substantially lower than the 58-fold increase reported for *Physcomitrella* [11]. The large discrepancy probably arises from differences in the uptake of P into biomass. Our measured values for the liverworts (7.3 µmol g^−1^ dwt tissue) were nearly five times lower than the values assumed (and not measured) for *Physcomitrella* (approx. 33 µmol g^−1^ dwt tissue) [11].

**Figure 3.**
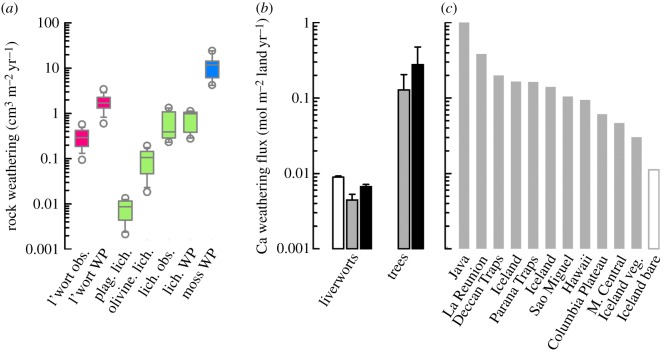
Comparative weathering by non-vascular plants, lichens and trees. (*a*) Rock volume weathering rates (median ± range and outliers) are derived from estimates of weathering potential (WP) calculated as a factor of annual plant-specific P requirement and the P content of basalt (after [9]). L'wort obs. refers to our observed Ca dissolution rates, which are converted to rock volume loss. plag. lich. and olivine lich. refer to observations of olivine and plagioclase mineral weathering in basalt lava flows of known maximum age colonized by lichens (after [24]). lich. obs. refers to direct observations of weathered rock volumes beneath lichen colonies of known maximum age [23]. (*b*) Calcium-silicate weathering fluxes associated with liverworts and trees grown under similar experimental conditions. Fluxes are scaled to 1 m^2^ of land based on the volume of soil affected by mycorrhizal hyphal networks. Open bar is 200 ppm CO_2_, grey bars are 450 ppm CO_2_ and black bars are 1200 ppm (liverworts) or 1500 ppm (trees) CO_2_. (*c*) Calcium weathering fluxes from major basaltic catchments (calculated from cation fluxes after [34]); Iceland veg. and Iceland bare refer to observed catchment-scale Ca fluxes from neighbouring unvegetated and small tree-covered basaltic catchments in Iceland, respectively [10].

A cross-check on our Ca-weathering rates can be made by defining them in terms of the volume of rock weathered over time and comparing these numbers with rates compiled from the literature (figure 3*a*). Calcium weathering rates in our liverwort experiments (l'wort obs. in figure 3*a*) fall within the range of available data and are higher than some estimates for lichens—algal–fungal partnerships that may have originated in the early Palaeozoic [35,36], and also weather exposed rock surfaces (figure 3*a*; electronic supplementary material, Detailed methods). Furthermore, reported weathering rates for both liverworts and lichens are consistent with modelled estimates derived from nutrients required to support contemporary net CO_2_ fixation rates by these organisms [9]. The weathering rate of mosses, as estimated from their biomass and P content, is approximately an order of magnitude higher than for liverworts or lichens (figure 3*a*). However, this weathering rate is probably exaggerated by moss adaptations for absorbing nutrients directly from rainfall, dust and leachates from vascular plant canopies [37]. Our data suggest thalloid liverworts weather basalt rock at a comparable rate with lichens (figure 3*a*).

In the field, the total flux of biologically weathered products reaching run-off depends, in part, on the depth of plant–substrate interactions. Deeper rooting systems enhance the total weathering flux by increasing mineral surface areas in contact with proliferating roots/rhizoids and symbiotic fungal hyphal networks. Deep roots anchor sediments and raise soil CO_2_ concentrations generated by respiration at depth to an extent that is proportional to NPP [28]. We, therefore, compare calculated weathering fluxes per unit land area for liverworts with those for trees in similar experimental systems and environmental conditions [2], after scaling for the effects of rhizoid/rooting depth to account for the volume of material being weathered. When our grain-scale weathering rates (electronic supplementary material, tables S2 and S3) are integrated with rhizoid/root and mycorrhizal hyphal density through the soil profile (figure 3*b*), total weathering per unit area of land under trees exceeds that of liverworts by factors of 5–14, to greater than 30, depending on growth CO_2_ concentration and mycorrhizal type. These scaled Ca weathering fluxes for trees are consistent with some estimates from vegetated watersheds with basaltic lithology, which display a variability reflecting additional effects of regional climate, relief and tectonic history (figure 3*b,c*) [10,34]. Furthermore, observations for Ca weathering fluxes from basalt beneath bryophytes and lichens corroborate the much lower weathering fluxes by the liverworts compared with trees. These comparisons indicate depth is a major limitation on the weathering capacity of the shallow rhizoid-based anchorage systems of early land plants [17].

Recent work proposing a major role for early land plants in driving mineral weathering and biogeochemical cycling of elements impacting climate in the Ordovician, extrapolated weathering rates at the grain-scale to global, land-to-ocean element fluxes. Using this approach, amplification of weathering by early land plants, relative to non-vegetated surfaces, was considered to be 25–75% of that ascribed to modern forests, which is a factor of approximately 6.7 [11,32]. The range reflects uncertainty in the effectiveness of weathering by early land plants relative to trees based on limited laboratory [11] and field [10] evidence. However, when rooting depths and soil volumes are considered, rootless non-vascular plants (weathering approx. 0.005–0.01 mol Ca m^−2^ yr^−1^) may only have achieved an amplification effect of approximately 2.5–5% that of contemporary trees (weathering approx. 0.2 mol Ca m^−2^ yr^−1^) on colonized land areas (figure 3*b*).

Geochemical modelling [11] suggests this limited effect would be insufficient to drive the dramatic atmospheric CO_2_ sequestration necessary for triggering glaciations, even given the collision-dominated interval of the Ordovician, which repeatedly rejuvenated continental surfaces with fresh silicate rocks and promoted weathering. So although the Taconic orogeny, which commenced in the Late Middle Ordovician, caused a long-term decline in atmospheric CO_2_ through increased exposure of fresh silicate rocks for weathering, alternative mechanisms triggering Ordovician glaciations appear necessary [38]. Additionally, the hypothesis that the origin and diversification of early terrestrial plants massively enhanced land-to-ocean P fluxes to fertilize the oceans and increase marine organic carbon burial [11] is not supported by our experiments. The rates of grain-scale P release by liverworts were lower than previous reports, and we show that the depth of plant-fungal-substrate interactions is crucial in scaling from weathering at the grain-scale to catchment-scale weathering fluxes. This constrains the abilities of early rootless plants to affect massive geochemical changes and suggests other mechanisms enhancing P delivery to the oceans might have operated during the Palaeozoic. For example, higher global temperatures combined with greater relative exposed area of crystalline apatite-bearing shield rocks could have generated a P weathering flux around twice that of today [29]. This could have been further supplemented by extensive Ordovician deposition of volcanic ash [30], a highly weatherable source of P.

## 4. Conclusion

We conclude that simple thalloid liverworts and their mycorrhizal symbionts increased basalt weathering rates at the grain-scale compared with plant-free controls through highly localized acidification (figures 1 and 2), to an extent similar to lichens (figure 3). After accounting for the depth of plant-fungal-substrate interactions, however, our experimental results for liverwort-driven weathering scale-up to yield weathering fluxes comparable with those reported from field studies of basaltic catchments colonized by lichens and bryophytes only (figure 3). Consequently, we suggest early land floras anchored into thin soils with shallow rhizoids and mycorrhizal partners probably exerted rather small effects on land-to-ocean fluxes of P and Ca–Mg and their associated role in atmospheric CO_2_ drawdown. Instead, the later evolutionary rise of rooted vascular plants and mycorrhizal fungi led to an increase in the capacity for biological interaction with rocks and minerals, which in turn caused an increase in the production of pedogenic clays. A pivotal point in this evolutionary progression was the rise in the productivity and biomass of Devonian forested ecosystems in upland regions. Forests with complex rooting systems substantially increased carbon-energy fluxes exported belowground [2] to intensify biological weathering processes through well understood mechanisms [3,32], with major effects on long-term CO_2_ and climate [21,32,39].

## Supplementary Material

Quirk et al Supporting Material

## Data accessibility

The data used in this paper are available from the Dryad data repository (http://dx.doi.org/10.5061/dryad.6dh6g).
