# Supplementary material for: Constraining the role of early land plants in Palaeozoic weathering and global cooling
Source: Proc Biol Sci. 2015 Aug 22;282(1813):20151115. doi: 10.1098/rspb.2015.1115 (PMC4632622; doi:10.1098/rspb.2015.1115)
Supplement: Quirk et al Supporting Material [file rspb20151115supp1.pdf]

Electronic Supporting Materials

Constraining the role of early land plants in Palaeozoic weathering and global cooling

Joe Quirk*, Jonathan R. Leake, David A. Johnson, Lyla L. Taylor, Loredana Saccone and David J. Beerling

Department of Animal and Plant Sciences, University of Sheffield, Sheffield, S10 2TN, UK

*email: j.quirk@sheffield.ac.uk

This document contains:

- Detailed Methods
- Figure S1
- Tables S1 – S3

NB. Reference numbers are independent of those in the main paper

Detailed Methods

Experimental details

Gametophytes of the thalloid liverwort, *Marchantia paleacea* (Bertol.) (from the phylogenetic sub-group, Marchantiopsida [1]) colonised by natural populations of mycorrhizal fungi were originally collected from a temperate cloud forest in Veracruz, Mexico [2]. Propagules from the dominant haploid gametophyte lifecycle stage were taken from established colonies kept in controlled-environment growth chambers. The presence or absence of mycorrhizal colonisation was manipulated by growing gametophytes either alongside mycorrhizal plants to ensure the formation of natural arbuscular mycorrhiza (AM)-like associations (M liverworts), or in isolation on substrate lacking mycorrhizal propagules to ensure non-mycorrhizal plants (NM liverworts) [2]. Genomic DNA extraction and sequencing from liverwort thallus fragments, described elsewhere [2, 3], confirmed the liverworts were partnered by Glomeromycotean fungi. We developed even-aged plants for the experiments, which were transplanted into experimental systems (Fig. S1a).

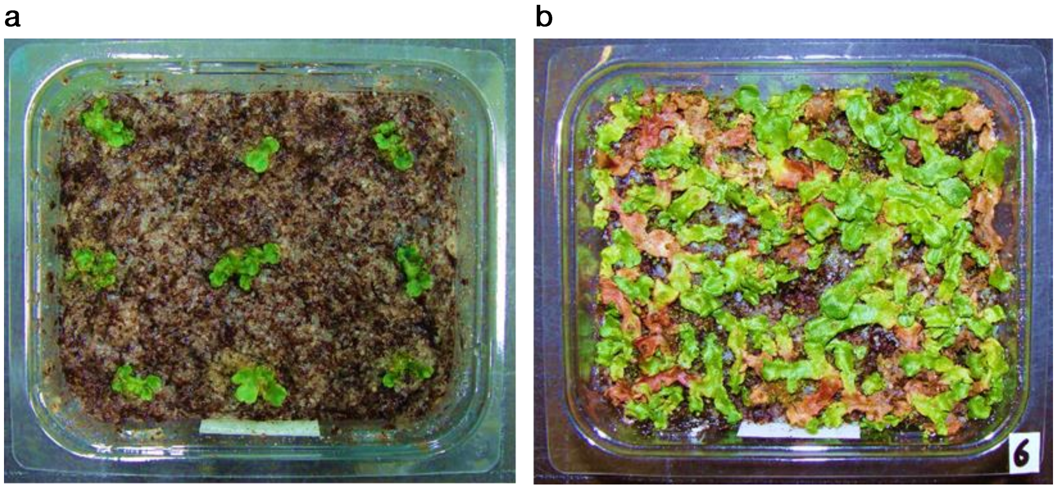

Figure S1. Liverwort colony at the beginning and (**a**) and after ~10 months (**b**). The trays are 114 × 86 mm.

We cultivated *M. paleacea* “colonies” in clear polyethylene terepthalate glycol (PETG) plant-culture containers (114 × 86 × 65 mm) (Phytatray™, Sigma-Aldrich) (Fig. S1) modified with four 12 mm-diameter mesh-covered drainage holes and eight 5 mm-diameter ventilation holes in the lids using heated cork borers. Approximately nine gametophytes (Fig. S1a) of M or NM *M. paleacea* were transplanted into the experimental containers on a substrate mixture of 2.7% (dry weight) *Sphagnum* peat collected from dune slacks in Anglesey, UK (5 g air-dried weight) and high-purity crushed quartz comprising three grain-size fractions (85g 106–250 µm; 38g 250–425 µm; and 57g 425–1000 µm). The depth of the substrate was ~45 mm. The concentration of total phosphorus in the bulk substrate was 0.37 µmol g^{-1} (determined from

aqua regia digestion). Liverwort colonies (n = 5) were cultivated alongside plant-free control treatments in controlled-environment growth chambers (Conviron BDR16, Conviron, Canada) at either 200 ppm, 450 ppm or 1200 ppm atmospheric CO_2, and otherwise constant environmental conditions of 70% relative humidity, 15/18°C night/day cycle with a 14-hr photoperiod of 80 $\mu mol\ m^{-2}\ s^{-1}$, which is about half light-saturating conditions for non-vascular plant groups [4]. All systems were rotated between cabinets on a monthly basis and misted with deionised water daily to maintain moisture of the foliage and substrate. We supplemented the liverworts with nitrogen in the form of 10 ml NH_4NO_3 solution (8 mg $NH_4NO_3\ L^{-1}$) fortnightly.

Basalt-filled hyphal in-growth mesh bags

Tertiary basalt from Northern Ireland (described previously [5]) was crushed in a ball mill, sieved into 0.25–1.00 mm grain sizes, rinsed with deionised water until the rinsate was visibly clear and dried at 50°C for 48 hr. The grains were weighed into 2.0 g portions and sealed into 50 × 50-mm right-angled triangle fine-mesh bags (35 µm pore-size woven nylon; Plastok Associates Ltd. Birkenhead, Wirral, UK) using a heat-sealer to melt and fuse the edges. The mesh permitted liverwort rhizoids, fungal hyphae and soil solutions to interact with basalt grains. X-ray fluorescence data for the basalt highlight its significance as a source of plant-essential macronutrients in our experiments (table S1). Two hyphal in-growth bags were buried side-by-side 1–2 cm below the soil surface before the experimental plants were transplanted. At the end of the experiment the bags were removed and rinsed in 10 ml of deionised water for 40 minutes (pH of solution determined) and dried at 50°C.

Hyphal lengths colonising basalt grains

A 1 g sub-sample from each mesh bag was sonicated for 10 min (Branson B32 sonic bath, Danbury CT, USA) in a 100 ml conical flask with 30 ml of deionised water to release adhering hyphal strands from the grains into suspension. Aliquots (4 ml) of the suspension were filtered through 25 mm diameter, 0.45 µm pore size gridded cellulose Whatman membrane filters. The retained hyphal strands were stained with 2 ml of Trypan blue lacto-phenol (0.4 g Trypan Blue ($C_{34}H_{24}N_6O_{14}S_4Na_4$), 50 g phenol ($C_6H_5OH$), 50 ml lactic acid ($C_3H_6O_3$), 100 ml glycerol ($C_3H_5(OH)_3$) and 50 ml deionised water) for 10 minutes. Excess stain was rinsed through the filter apparatus with deionised water and hyphal strands were measured via line-intersect counts at 200× magnification [6].

Assessing physical mineral surface alteration

We used Mirau vertical scanning interferometry (VSI) in white light mode to characterise the surfaces of mineral flakes before and after incubation in liverwort experimental systems,

following ref. [7]. Each VSI scan produces an *XY* data array where each pixel corresponds to surface height *(Z)* relative to the mean surface plane, where *Z* is equal to zero. Flakes (~4 × 4 mm) of phlogopite (Iveland, Norway) and biotite (Moen, Norway) were embedded in silicone (commercially available silicone sealant) and mounted on 26 × 4 mm glass 'VSI slides', as previously described [7].

Phlogopite and biotite are Mg- and Mg- and Fe-bearing Al-phyllosilicates, respectively with perfect basal cleavage making them ideal minerals for investigations of surface alteration with VSI. Prior to burial, the surface of each mineral flake was cleaned first using ~200 μl 1% sodium dodecyl sulphate (SDS) and lens tissue, followed by lens tissue wetted with deionised water [8]. The VSI slides were mounted on an automated stage platform and randomly selected localities (500× mag., 126 × 94 μm scanned area) on the surface of each mineral flake were scanned using a Wyko NT9100 VSI instrument (Bruker AXS, Madison WI, USA) positioned on a vibration-minimising air table. We obtained the root mean square (RMS) roughness [8] of each surface location (2 surface locations on each mineral flake) using the instrument's analytical software (Vision 4.10, Bruker AXS, Madison WI, USA). The location of each scan has known stage coordinates that, in combination with an automated platform and VSI slide mount, allow precise relocation and re-characterisation of surface topography at each scan location after incubation in the experimental treatments [7].

The VSI slides were co-buried along with the crushed basalt grains within the mesh bags. At the end of the experiment, VSI slides were recovered, re-cleaned with 1% SDS followed by deionised water, and their RMS roughness re-measured. We took the ratio of the post-experiment and pre-experiment RMS roughness as a metric of mineral surface alteration over the course of the experiment, including plant-free control treatments, where values >1 indicate surface roughness increases following physical alteration. We background-corrected the roughness ratios using the values obtained from the plant-free control treatments, where roughness increases are associated with soil pore waters and free-living populations of microorganisms [7]. Roughness ratios were averaged to give one value per mineral type, per replicate liverwort colony (n = 5 colonies)

Fungal trenching of mineral surfaces

Each mineral flake was visually analysed for evidence of fungal hyphal trenching using the live camera feed on the VSI instrument. Where trenches were observed, width and depth measurements were carried out in Vision 4.10. Two-dimensional transects were analysed at 10-μm intervals at right-angles to the linear trenches allowing assessment of the width at the top of the trench and maximum depth relative to the surrounding planar surface. Trench X-sectional areas were defined on the basis of the area of a triangle, as previously described [7]. Where

trenches were observed, we obtained mean trench dimensions for each mineral type within each individual colony (*n* = 3 – 5 colonies).

Element uptake into liverwort biomass

We used an aqua regia tissue digest with 3:1 $HCl:HNO_3$ at 150°C for 2 hr to assess elemental uptake into liverwort biomass, and phosphorus concentrations in the bulk substrate. Using acid-washed glassware, we digested ~25 mg of homogenised thallus tissue by ramping the temperature in 5°C intervals from 60°C to 150°C and maintained for 2 hr. Residues were filtered through 0.20 µm syringe filters (Minisart High-Flow, Sartorius, Goettingen, Germany) and diluted to 14 ml with ultra-high purity water. The concentration of Ca was determined using inductively coupled plasma mass spectrometry (ICP-MS) (PerkinElmer Elan DRC II, MA, USA). Phosphorus uptake into thallus material was determined by digesting 25 mg of homogenised thallus tissue in 1 ml H_2SO_4 (96% w/w, 18 mol l^{-1}) at 365°C. After 25 min, 100 µl H_2O_2 (30%) was successively added until digest solutions turned clear. Digest residues were diluted 1:40 with deionised water and thoroughly mixed. Phosphorus concentrations in digest solutions were quantified spectophotometrically at 882 nm λ (Cecil C120 spectrophotometer, Cambridge, UK) using a standard ascorbic acid and molybdate colour-development assay calibrated against a dilution series of laboratory P standards (K_3PO_4) spanning the range of concentrations in sample tissues.

Grain-scale calcium and phosphate dissolution from basalt

All the weathering rates we report from basalt in the experiments are relative to reference basalt grains, not absolute rates. The reference grains are either freshly prepared, unweathered grains from the same batch of basalt used in the experiments, or grains that have been recovered from plant-free treatments. We assessed calcium dissolution using a sequential extraction protocol described previously [5]. Briefly, the extractions sequentially remove the water-extractable, exchangeable (1M ammonium acetate), carbonate (1M sodium acetate and acetic acid, pH 5.0) and oxide fractions (0.5M hydroxylamine-hydrochloride in 25% acetic acid followed by 0.1M ammonium oxalate adjusted to pH 3.0 with 0.2M oxalic acid and 0.1M ascorbic acid) of the basalt grains. Analyses demonstrate that following the extraction of Ca from the exchangeable and carbonate phases, Ca extracted thereafter is predominantly derived from dominant silicate minerals within the basalt (table S1) [5]. Extraction solutions were filtered (0.20 µm), diluted, acidified with 1% HNO_3 and Ca concentrations of the nominal carbonate and oxide (silicate) fractions were determined using ICP-MS (PerkinElmer Elan DRC II). We quantified the P concentration in each extraction solution using an ascorbic acid and molybdate colour-development assay, as above. Weathered amounts of Ca and P from basalt (µmol) at the grain-

scale were calculated as the difference between extractable Ca or P from the carbonate (f1) and oxide (silicate) fractions (f2) of fresh, unweathered basalt samples ($_{UW}$) and that extracted from the same mineralogical phases from the basalt grains in the liverwort and plant-free experimental treatments ($_{treat}$) (eq.S1):

$$Dissolution = (f1_{UW} + f2_{UW}) - (f1_{treat} + f2_{treat}) \qquad \text{eq.S1}$$

We accounted for uptake of weathered elements into liverwort tissues and calculated weathering amplification factors as the ratio of dissolution values between liverworts plus tissue contents and plant-free treatments.

Comparison of liverwort weathering rates with that of trees

We used basalt dissolution data from a previous set of biological weathering experiments using exemplar taxa spanning major phases of evolutionary diversification in tree and mycorrhizal fungal functional types (gymnosperm versus angiosperm and AM fungi versus more recently evolved ectomycorrhizal [EM] fungi) [9]. This experiment utilised gymnosperms in association with both AM fungi (*Ginkgo biloba* and *Sequoia sempervirens*) and EM fungi (*Pinus sylvestris*); and angiosperms in association with both mycorrhizal types (*Magnolia grandiflora* – AM, and *Betula pendula* – EM). Saplings were sourced from UK nurseries and cultivated in 160 mm-diameter, 1800 mm-tall polyvinylchloride free-draining weathering reactors containing 2:1 by volume quartz sand and compost. The substrate was mixed with natural mycorrhizal inoculum consisting of species-specific fresh root material sourced from the National Arboretum, Westonbirt, UK. Saplings (n = 4) were kept alongside plant-free reactors in replicated controlled-environment growth rooms (Conviron, Controlled Environments Ltd. Manitoba, Canada) (two per CO_2) and maintained at 450 ppm or 1500 ppm CO_2 at 70% relative humidity, 18/20°C night/day cycle with a 14-h photoperiod. See Quirk *et al.* [9] for details.

Hyphal in-growth cores constructed from 85 mm-length polyvinylchloride pipe (13.4 mm-diameter) with windows of ~2500 mm^2 total area covered in root-excluding nylon mesh (35 µm-pore-size) were inserted horizontally into ports at 200 mm-depth in the side of the weathering reactors. Cores were filled with 5.0 g of the basalt (0.3–2.0 mm grain size) described above, along with 4.0 g of 0.05–0.10 mm pure quartz sand. We assessed calcium dissolution at the end of the experiment using the sequential chemical extraction procedure and ICP-MS to quantify calcium concentrations. Because the previously published datasets for tree-driven calcium weathering rates were expressed relative to the dissolution rate in plant-free controls, not freshly prepared, unweathered basalt grains [9], we calculated calcium weathering rates for both the trees and liverworts for each treatment relative to basalt from plant-free treatments using (eq.S2):

$$Dissolution\ rate = [(f1_{pf} + f2_{pf}) - (f1_{plant} + f2_{plant})] / time \qquad \text{eq.S2}$$

where the subscript '$_{pf}$' is the amount of Ca extracted from basalt in plant-free treatments of either the trees or liverworts (μmol g^{-1}), '$_{plant}$' is the amount of Ca extracted from basalt in each replicate tree or liverwort treatment (μmol g^{-1}) and *time* is the duration of the experiment (years). Note the main difference between eq.S1 and eq.S2 is whether the reference dissolution value is taken from freshly prepared, unweathered basalt grains (eq.S1) or those that have been weathered to relatively small 'background' rates in plant-free treatments (eq.S2), where they have been influenced by soil pore waters and free-living communities of soil bacteria and fungi. These grain-scale basalt weathering rates are presented in tables S2 and S3.

Scaling grain-scale dissolution rates to total weathering fluxes by accounting for rooting depth

For the liverworts, we defined the volume of soil under the influence of mycorrhizal fungal hyphae – the hyphosphere – for each plant type [10]. We determined hyphospheres for the liverwort treatments using a standardised hyphal diameter of 2.8 μm and a representative hyphal length density of 5m cm^{-3} soil based on published values and previous model parameterisation [11]. We re-plotted hyphal length density data from Jakobsen *et al.* [12] against distance from host roots of *Trifolium subterraneum* seedlings growing in a comparable sand-based, phosphorus-poor substrate to that used here. A linear length density decay function – y (m cm^{-3}) = -0.983x + 11.569 – fitted to these data established that AM hyphal length densities were zero by 12 cm, considered here to be the maximum depth of AM hyphal growth from liverwort thalli. We normalised the hyphal length densities colonising basalt grains against the estimated upper length in our modelled bulk soil volume (based on a hyphal density of 5m cm^{-3}) and calculated the hyphosphere volume (m^3) using: $l \cdot \pi \cdot (H_{sphere}^2 - H^2)$, where H_{sphere} and H are the radii of the hyphosphere and hyphae, respectively. Consequently, the individual "colony" with the highest hyphal length density associated with basalt grains in the current study was assigned the upper bulk substrate hyphal length value (588 km m^{-2}) based on the dimensions of the containers used, a modelled soil volume of at least 12 cm depth and a hyphal density in soil of 5 m cm^{-3}. This means our scaled liverwort dissolution estimates likely represent upper values. We then defined the hyphosphere surrounding the basalt grains and scaled-up weathering rates per unit hyphosphere to a weathering flux for the total hyphosphere volume beneath 1 m^2 of liverwort-colonised land (mean = 4.4×10^{-5} m^3 m^{-2}).

For the trees we defined the distribution of fine roots and mycorrhizal hyphae in soils beneath 1 m^2 of forested land, as represented by the tree taxa, following the assumptions of Taylor *et al.* [11, 13]. The approach is based on the exponential extinction of fine root distribution with depth for representative plant and mycorrhiza functional types [11, 14, 15]. The procedure, described in detail elsewhere [11, 13], uses a characteristic soil profile depth, which,

when multiplied by the hyphal length density at the top of the soil profile for both representative AM (5 m cm^{-3} soil) [11, 16] and EM trees (250 m cm^{-3} soil) [6] gives the integrated, total length of hyphae in the soil profile per m^2 of land. The characteristic depth is derived from area-averaged net primary productivity (NPP) (g C m^{-2} yr^{-1}) for specific tree types [17], which is linearly related to characteristic rooting, and, by extension, mycorrhizal hyphal depth [11]. Using integrated hyphal lengths, we determined hyphosphere volumes for the trees of 0.00303 m^3 m^{-2}.

Using previously published values of AM and EM hyphal colonisation of basalt grains [5] associated with a range of tree functional types (2–10 m hyphae g^{-1} basalt grains), we were able to calculate hyphosphere volumes associated with the basalt grains for the different trees. We scaled the calcium dissolution rates from basalt grains to the scale of the hyphosphere per m^2 land for both liverworts and trees – that is the total flux of weathered calcium – assuming a soil calcium mole fraction of 0.5% (after Moulton *et al.* [18] and Blum *et al.* [19]).

Comparisons of potential and observed weathering between liverworts, lichens and moss

We verified the estimates for liverwort weathering by calculating potential weathering rates for the liverworts, as well as mosses and lichens. Potential weathering is an estimate of weathered rock volume and is derived as a function of the annual plant P requirement for a given area of non-vascular plant cover, and the P concentration of the rock type the plants live and depend upon for P-uptake (in this case the basalt, which has a P concentration of 3.77 mg P cm^{-3}), following Porada *et al.* [20]. We use a basalt density of 2.9 g cm^{-3}, after Porada *et al.* [20]. Plant P requirement is adjusted by accounting for a biological re-sorption rate of 50% and a pre-uptake leaching factor of 35% in accordance with Porada *et al.* [20]. Although this method only estimates weathering rates, it offers a means for comparing and contextualising our and others' observed weathering rates for non-vascular plants and lichens against theoretical process-based models [20]. We assume the liverworts in our study were reliant on the basalt for P-uptake (whilst accounting for P leaching and re-sorption from decaying organic material) and expressed the potential weathering rate for liverworts, lichens and moss as the volume of rock weathered.

To calculate potential weathering we needed the specific mass and annual P requirement of the liverworts, lichens and moss, which, if not reported in the literature, were calculated from estimates of yearly growth. Phosphorus requirements and specific masses for the liverworts were calculated from the present study. We defined mass-area ratios for the liverworts using dry mass at the end of the experiment and calculated area of coverage using ImageJ (open source, National Institutes of Health, Bethesda, Maryland, USA) with digital photographs taken of the liverwort colonies a few days before they were harvested. We determined P requirements for the liverworts based on their P contents and a growth rate of 6.7

± 0.5 mg m^{-2} yr^{-1}. Specific masses of lichens were taken from Gauslaa & Solhaug [21], Hilmo [22], Palmqvist & Sundberg [23] and Asplund *et al.* [24], with lichen P contents taken from Chapin & Shaver [25] and Hogan *et al.* [26]. Lichen growth analysis from Karlén and Black [27] was used to calculate estimated annual P requirements of 3.1 ± 0.3 mg P m^{-2} yr^{-1}. Moss specific mass and growth per annum were established from Lenton *et al.* [28], Horsley *et al.* [29], Ino and Nakatsubo [30] and Fenton [31], and annual P requirement was based on a standardised moss tissue P concentration of 0.3 mg g^{-1} tissue [28, 30], giving 43 ± 8 mg P m^{-2} yr^{-1}.

We cross-checked the weathering potential rates for the liverworts (calculated from their annual P requirements for growth) by estimating the volume of rock weathered from the basalt based on our observations of Ca dissolution from the basalt grains (mol Ca g^{-1} rock yr^{-1}), the concentration of Ca in the basalt (5.39 mmol Ca cm^{-3}) and the density of the basalt (2.9 g cm^{-3}). This estimate is referred to as *L'wort obs.* in figure 3a. We also found evidence with which to ground-truth the weathering potential estimates from observational studies reporting lichen weathering rates in terms of the volumetric loss of rock material beneath lichens relative to un-vegetated surfaces. In figure 3a, *Olivine lich.* and *Plag. lich.* refer to visual observations reported by Brady *et al.* [32] of olivine and plagioclase mineral weathering (defined as percentage mineral porosity increase per Kyr) in basalt lava flows of known age with or without lichen colonisation. We converted mineral porosity increase into annual mass loss per unit area using representative specific gravities for olivine (3.8 g cm^{-3}) and plagioclase (2.7 g cm^{-3}) taken from Klein [33] and the average depth of the weathering rind beneath the lichens – given as 50 µm [32]. We then estimated annual basalt volume loss using the relative proportions of plagioclase and olivine in the basalt (table S1). *Lichen obs.* in figure 3a refers to direct observations of weathered rock volumes beneath lichen colonies of known maximum age, reported by McCarroll and Viles [34].

Verification of scaled calcium weathering fluxes for liverworts and trees against catchment-scale estimates

We cross-checked the scaled Ca weathering fluxes per m^2 beneath liverworts and trees against observations of weathering fluxes from vegetated catchments, underlain by basaltic lithologies, across the globe [18, 35]. Fluxes of dissolved cations (Na+K+Ca+Mg), reported by Dessert *et al.* [35], were used to estimate Ca weathering fluxes based on the stoichiometry of major oxides in basalt (Table S1), and should therefore be regarded as close approximations. Calcium weathering fluxes reported by Moulton *et al.* [18] for neighbouring Icelandic catchments of the same basaltic lithology (one of which was vegetated by small trees [0.011 mol m^{-2} yr^{-1}] and the other by non-vascular bryophytes and lichens only [0.030 mol m^{-2} yr^{-1}]) were converted to estimates of volumetric basalt weathering rates (cm^3 m^{-2} yr^{-1}). This was approximated using a

basalt density of 2.9 g cm^{-3} and calcium concentration of 5.4 mmol Ca cm^{-3} in order to ground-truth the estimates of rock weathering potential reported by Porada *et al*. [20] from numerical modelling of lichen and bryophyte carbon assimilation and rock weathering.

Table S1. Concentrations of major and trace elements in the basalt grains (determined using XRF). Taken from Quirk *et al.* [5] CIPW normative mineral composition was calculated from the normalised weight % of the 11 major oxides using the Excel program* freely provided by Prof. Kurt Hollocher, Geology Department, Union College, Schenectady, NY, USA. Basalt specific gravity is ~2.9 g cm^{-3}.

Major oxides	XRF analysis wt%	Normalised wt%	Trace elements	ppm	Normative minerals	Normalised wt%
SiO_2	44.22	41.9	Rb	3.7	Quartz	-
TiO_2	0.78	0.74	Sr	252.7	Plagioclase	50.19
Al_2O_3	17.05	16.16	Nb	2.1	Orthoclase	1.71
FeO	10.51	9.96	Zr	64.4	Diopside	9.32
Fe_2O_3	11.93	11.3	Y	24.2	(Mg)(Fe)SiO_3 Opx.	14.49
MnO	0.15	0.15	Pb	-	Olivine	5.89
MgO	8.10	7.68	Ga	16.8	Ilminite	1.41
CaO	10.37	9.82	Cr	255.9	Magnetite	16.38
Na_2O	1.84	1.75	Ni	530.8	Apatite	<u>0.63</u>
K_2O	0.30	0.29	V	199.5	Sum	100.02
P_2O_5	0.29	<u>0.27</u>	Zn	67.2		
	Sum	100.02	Ba	232.7		
			La	11.8		
			Ce	18.4		
			Nd	10.8		
			Yb	1.1		

*(http://minerva.union.edu/hollochk/c_petrology/norms.htm)

Table S2. Grain-scale dissolution rates of calcium from basalt incubated beneath arbuscular mycorrhizal (AM) and non-mycorrhizal (NM) *Marchantia paleacea* liverworts under laboratory conditions and controlled atmospheric CO_2 concentrations. Units are μmol g^{-1} basalt yr^{-1} with s.e.m. in parentheses (*n*=5). Dissolution rates are relative to plant free treatments.

	Calcium dissolution (μmol g^{-1} yr^{-1})		
	Atmospheric CO_2 concentration		
	200 ppm	450 ppm	1200 ppm
AM	23.5 (1.3)	13.9 (1.9)	10.8 (1.0)
NM	15.9 (3.4)	14.8 (1.0)	13.4 (1.6)

Table S3. Grain-scale dissolution rates of calcium from basalt incubated beneath arbuscular mycorrhizal (AM) and ectomycorrhizal (EM) trees under laboratory conditions and controlled atmospheric CO_2 concentrations. Units are μmol g^{-1} basalt yr^{-1} with s.e.m. in parentheses (*n*=4). Dissolution rates are relative to plant free treatments.

Species (mycorrhiza type)	Calcium dissolution (μmol g^{-1} yr^{-1})	
	Atmospheric CO_2 concentration	
	450 ppm	1500 ppm
Ginkgo biloba (AM)	54.4 (7.9)	27.1 (11.4)
Sequoia sempervirens (AM)	16.8 (10.3)	34.3 (10.9)
Magnolia grandiflora (AM)	56.7 (9.2)	25.8 (5.8)
Pinus sylvestris (EM)	37.4 (16.8)	62.6 (5.8)
Betula pendula (EM)	96.5 (23.3)	61.4 (14.7)

Additional References

[1] He-Nygrén, X., Juslén, A., Ahonen, I., Glenny, D. & Piippo, S. 2006 Illuminating the evolutionary history of liverworts (Marchantiophyta)—towards a natural classification. *Cladistics* **22**, 1-31. (doi:10.1111/j.1096-0031.2006.00089.x).

[2] Humphreys, C.P., Franks, P.J., Rees, M., Bidartondo, M.I., Leake, J.R. & Beerling, D.J. 2010 Mutualistic mycorrhiza-like symbiosis in the most ancient group of land plants. *Nature Commun.* **1**. (doi:10.1038/ncomms1105).

[3] Field, K.J., Cameron, D.D., Leake, J.R., Tille, S., Bidartondo, M.I. & Beerling, D.J. 2012 Contrasting arbuscular mycorrhizal responses of vascular and non-vascular plants to a simulated Palaeozoic CO_2 decline. *Nature Commun.* **3**, 835-835. (doi:10.1038/ncomms1831).

[4] Nobel, P.S. 2009 *Physicochemical and environmental plant physiology*. 4th ed. Amsterdam ; London, Academic.

[5] Quirk, J., Beerling, D.J., Banwart, S.A., Kakonyi, G., Romero-Gonzalez, M.E. & Leake, J.R. 2012 Evolution of trees and mycorrhizal fungi intensifies silicate mineral weathering. *Biol. Lett.* **8**, 1006-1011.

[6] Wallander, H., Göransson, H. & Rosengren, U. 2004 Production, standing biomass and natural abundance of ^{15}N and ^{13}C in ectomycorrhizal mycelia collected at different soil depths in two forest types. *Oecol.* **139**, 89-97.

[7] Quirk, J., Leake, J.R., Banwart, S.A., Taylor, L.L. & Beerling, D.J. 2014 Weathering by tree-root-associating fungi diminishes under simulated Cenozoic atmospheric CO_2 decline. *Biogeosciences* **11**, 321-331. (doi:10.5194/bg-11-321-2014).

[8] Buss, H.L., Lüttge, A. & Brantley, S.L. 2007 Etch pit formation on iron silicate surfaces during siderophore-promoted dissolution. *Chem. Geol.* **240**, 326-342.

[9] Quirk, J., Andrews, M.Y., Leake, J.R., Banwart, S.A. & Beerling, D.J. 2014 Ectomycorrhizal fungi and past high CO_2 atmospheres enhance mineral weathering through increased below-ground carbon-energy fluxes. *Biol. Lett.* **10**. (doi:10.1098/rsbl.2014.0375).

[10] Marschner, H. 1995 *Mineral nutrition of higher plants*. 2nd ed. London, Academic.

[11] Taylor, L.L., Banwart, S.A., Leake, J.R. & Beerling, D.J. 2011 Modeling the evolutionary rise of ectomycorrhiza on sub-surface weathering environments and the geochemical carbon cycle. *Am. J. Sci.* **311**, 369-403. (doi:10.2475/05.2011.01).

[12] Jakobsen, I., Abbott, L.K. & Robson, A.D. 1992 External Hyphae of Vesicular-Arbuscular Mycorrhizal Fungi Associated with Trifolium subterraneum L. 1. Spread of Hyphae and Phosphorus Inflow into Roots. *New Phytol.* **120**, 371-379.

[13] Taylor, L.L., Banwart, S.A., Valdes, P.J., Leake, J.R. & Beerling, D.J. 2012 Evaluating the effects of terrestrial ecosystems, climate and carbon dioxide on weathering over geological time: a global-scale process-based approach. *Philos. Trans. R. Soc. B-Biol. Sci.* **367**, 565-582. (doi:10.1098/rstb.2011.0251).

[14] Jackson, R.B., Canadell, J., Ehleringer, J.R., Mooney, H.A., Sala, O.E. & Schulze, E.D. 1996 A global analysis of root distributions for terrestrial biomes. *Oecol.* **108**, 389-411. (doi:10.2307/4221432).

[15] Jackson, R.B., Mooney, H.A. & Schulze, E.-D. 1997 A global budget for fine root biomass, surface area, and nutrient contents. *Proc. Natl. Acad. Sci. U.S.A.* **94**, 7362-7366.

[16] Ingleby, K., Diagne, O., deans, J.D., Lindley, D.K., Neyra, M. & Ducousso, M. 1997 Distribution of roots, arbuscular mycorrhizal colonisation and spores around fast-growing tree species in Senegal. *Forest Ecology and Management* **90**, 19-27. (doi:10.1016/S0378-1127(96)03875-3).

[17] Saugier, B., Roy, J. & Mooney, H.A. 2001 Estimations of global terrestrial productivity: converging toward a single number. In *Terrestrial global productivity* (eds. J. Roy, B. Saugier & H.A. Mooney), pp. 543-557. San Diego, Academic Press.

[18] Moulton, K.L., West, J. & Berner, R.A. 2000 Solute flux and mineral mass balance approaches to the quantification of plant effects on silicate weathering. *Am. J. Sci.* **300**, 539-570. (doi:10.2475/ajs.300.7.539).

[19] Blum, J.D., Klaue, A., Nezat, C.A., Driscoll, C.T., Johnson, C.E., Siccama, T.G., Eagar, C., Fahey, T.J. & Likens, G.E. 2002 Mycorrhizal weathering of apatite as an important calcium source in base-poor forest ecosystems. *Nature* **417**, 729-731.

[20] Porada, P., Weber, B., Elbert, W., Pöschl, U. & Kleidon, A. 2014 Estimating impacts of lichens and bryophytes on global biogeochemical cycles. *Global Biogeochem. Cycles* **28**, 71-85. (doi:10.1002/2013GB004705).

[21] Gauslaa, Y. & Solhaug, K.A. 1998 The Significance of Thallus Size for the Water Economy of the Cyanobacterial Old-Forest Lichen Degelia plumbea. *Oecol.* **116**, 76-84. (doi:10.2307/4222060).

[22] Hilmo, O. 2002 Growth and morphological response of old-forest lichens transplanted into a young and an old Picea abies forest. *Ecography* **25**, 329-335. (doi:10.1034/j.1600-0587.2002.250309.x).

[23] Palmqvist, K. & Sundberg, B. 2000 Light use efficiency of dry matter gain in five macro-lichens: relative impact of microclimate conditions and species-specific traits. *Plant, Cell & Environment* **23**, 1-14. (doi:10.1046/j.1365-3040.2000.00529.x).

[24] Asplund, J., Sandling, A. & Wardle, D.A. 2012 Lichen Specific Thallus Mass and Secondary Compounds Change across a Retrogressive Fire-Driven Chronosequence. *PLoS ONE* **7**, e49081. (doi:10.1371/journal.pone.0049081).

[25] Chapin, F.S., III & Shaver, G.R. 1988 Differences in carbon and nutrient fractions among Arctic growth forms. *Oecol.* **77**, 506-514. (doi:10.2307/4218811).

[26] Hogan, E.J., Minnullina, G., Smith, R.I. & Crittenden, P.D. 2010 Effects of nitrogen enrichment on phosphatase activity and nitrogen : phosphorus relationships in *Cladonia portentosa. New Phytol.* **186**, 911-925. (doi:10.1111/j.1469-8137.2010.03222.x).

[27] Karlén, W. & Black, J.L. 2002 Estimates of lichen growth-rate in northern Sweden. *Geografiska Annaler* **84**, 225-232. (doi:10.2307/3566140).

[28] Lenton, T.M., Crouch, M., Johnson, M., Pires, N. & Dolan, L. 2012 First plants cooled the Ordovician. *Nature Geosci.* **5**, 86-89. (doi:10.1038/ngeo1390).

[29] Horsley, K., Stark, L.R. & McLetchie, D.N. 2011 Does the silver moss *Bryum argenteum* exhibit sex-specific patterns in vegetative growth rate, asexual fitness or prezygotic reproductive investment? *Annals of Botany* **107**, 897-907. (doi:10.1093/aob/mcr027).

[30] Ino, Y. & Nakatsubo, T. 1986 Distribution of carbon, nitrogen and phosphorus in a moss community-soil system developed on a cold desert in Antarctica. *Ecol. Res.* **1**, 59-69. (doi:10.1007/BF02361205).

[31] Fenton, J.H.C. 1980 The rate of peat accumulation in Antarctic moss banks. *J. Ecol.* **68**, 211-228. (doi:10.2307/2259252).

[32] Brady, P.V., Dorn, R.I., Brazel, A.J., Clark, J., Moore, R.B. & Glidewell, T. 1999 Direct measurement of the combined effects of lichen, rainfall, and temperature on silicate weathering. *Geochim. Cosmochim. Acta* **63**, 3293-3300. (doi:10.1016/S0016-7037(99)00251-3).

[33] Klein, C., Hurlbut, C.S. & Dana, J.D. 2002 *The 22nd edition of the manual of mineral science: (after James D. Dana)*. 22nd ed. New York ; Chichester, Wiley.

[34] McCarroll, D. & Viles, H. 1995 Rock-weathering by the lichen *Lecidea auriculata* in an arctic alpine environment. *Earth Surface Processes and Landforms* **20**, 199-206. (doi:10.1002/esp.3290200302).

[35] Dessert, C., Dupre, B., Gaillardet, J., Francois, L.M. & Allegre, C.J. 2003 Basalt weathering laws and the impact of basalt weathering on the global carbon cycle. *Chem. Geol.* **202**, 257-273. (doi:10.1016/j.chemgeo.2002.10.001).
